# Comparative evaluation of hrHPV DNA testing, cervical cytology and histopathology for cervical precancer detection

**DOI:** 10.6026/973206300223395

**Published:** 2026-06-30

**Authors:** Atul Jain, Mamta Meena, Shiny Vincent, Iqra Sheikh, Surabhi Sharma, Kuldeep Singh, Rajdeep Paul

**Affiliations:** 1 Department of Pathology, Bundelkhand Medical College, Sagar, Madhya Pradesh, India; 2 Department of Microbiology, Gandhi Medical College, Bhopal, Madhya Pradesh, India; 3 Department of Medical Laboratory, Leonard Hospital, Tamil Nadu, India; 4 Department of Clinical Pathology, Lok Nayak Hospital, New Delhi, India; 5 Department of Microbiology, Teerthanker Mahaveer Medical College & Research Centre, Moradabad, Uttar Pradesh, India; 6 Department of Microbiology, Chirayu Medical College & Hospital, Bhopal, Madhya Pradesh, India

**Keywords:** Cervical cancer, hrHPV DNA testing, cytology, histopathology, screening, genotyping

## Abstract

Cervical cancer remains a major public health challenge in India, driven primarily by persistent high-risk HPV infection. Hence, this prospective study evaluated hrHPV DNA testing, cervical cytology and co-testing in 400 symptomatic women, using histopathology as the gold standard for CIN2+ detection. HPV DNA testing showed the highest sensitivity (90%) and diagnostic accuracy (63.8%) compared with cytology and co-testing, while ROC analysis confirmed its superior performance (AUC 0.75). HPV16 was the predominant genotype, followed by HPV18, HPV52 and HPV58, together accounting for most high-grade lesions. Thus, we show HPV DNA testing as a more reliable primary screening method than cytology in resource-limited settings.

## Background:

Cervical cancer is the fourth most common cancer in women worldwide. In 2022, there were about 660,000 new cases and 350,000 deaths [1]. India carries a heavy burden, reporting 127,526 new cases and 79,906 deaths in the same year [2]. This makes cervical cancer one of the leading causes of cancer deaths among Indian women. The main cause of cervical cancer is long-term infection with high-risk types of human papillomavirus (hrHPV) [3, 4]. Because of this, HPV DNA testing has become a strong tool for finding precancer and cancer at an early stage. Globally, HPV16 and HPV18 are the most dangerous genotypes, together causing about 70% of cervical cancers [5]. Other common high-risk types such as HPV31, 33, 45, 52 and 58 also play an important role, especially in Asia, including India [6]. Knowing the local pattern of hrHPV genotypes is useful because it can guide both vaccination and screening programs. For many years, cervical cytology (Pap smear or liquid-based cytology, LBC) has been the main screening method. But cytology has limits. Its sensitivity is not very high and the results can vary depending on the person reading the slides [7]. In contrast, histopathology from a biopsy is still the gold standard. It is used to confirm and grade cervical lesions such as cervical intraepithelial neoplasia (CIN) [8]. Research has shown that HPV testing finds more high-grade precancer (CIN2+ or CIN3+) than cytology alone [9]. However, using HPV as the first screening test can lead to more women being sent for colposcopy, especially in the first round of screening [10]. This is a challenge for hospitals in places where resources are limited. In Central India, where access to advanced diagnostic tools is often poor, comparing the performance of these screening tests is very important. Therefore, it is of interest to compare the diagnostic performance of hrHPV DNA testing, cervical cytology, and co-testing against histopathology for the detection of cervical precancer.

## Materials and Methods:

## Study design and setting:

This was a prospective observational study conducted between January 1, 2024 and December 31, 2024. Cervical samples were collected from symptomatic women attending multiple collaborating centres across India. Molecular testing for high-risk HPV DNA was performed at the Department of Microbiology, CMCH, Bhopal, while histopathological evaluation was conducted at the Department of Pathology, BMC, Sagar. This multi-institutional collaboration integrated clinical recruitment, molecular testing and pathological expertise, while ensuring standardized procedures across all sites.

## Participants:

A total of 400 symptomatic women aged 25-65 years were enrolled. Inclusion criteria included abnormal vaginal discharge, post-coital bleeding, intermenstrual bleeding, or an unhealthy-appearing cervix. Exclusion criteria were pregnancy, prior hysterectomy, active vaginal infection, or previous treatment for cervical lesions. Sample size was calculated assuming a CIN2+ prevalence of 10-15%, providing adequate precision for sensitivity estimation.

## Sample collection and transportation:

Cervical samples were collected using cytobrushes and placed in liquid-based cytology (LBC) medium. Samples were stored at 2-8°C and transported within 24 to 48 hours to CMCH, Bhopal for HPV DNA testing, maintaining strict cold-chain protocols to preserve nucleic acid integrity. Colposcopy-guided biopsy specimens were fixed in 10% neutral-buffered formalin at the collection site and transported under standard conditions to BMC, Sagar. All transport and handling procedures followed standardized protocols to ensure sample quality.

## Procedures:

## Cervical cytology:

Samples were processed as LBC using the SurePath system. Slides were stained and reported according to the 2014 Bethesda System:

[1] Negative for intraepithelial lesion or malignancy (NILM)

[2] Atypical squamous cells of undetermined significance (ASC-US)

[3] Low-grade squamous intraepithelial lesion (LSIL)

[4] High-grade squamous intraepithelial lesion (HSIL)

[5] Squamous cell carcinoma (SCC)

## HPV DNA testing:

Residual LBC fluid was analyzed at CMCH, Bhopal using the TRUPCR® HPV High Risk Genotyping Kit (3B BlackBio Biotech India Ltd., India), a real-time PCR assay detecting 14 high-risk HPV types (16, 18, 31, 33, 35, 39, 45, 51, 52, 56, 58, 59, 66, 68) targeting E6/E7 regions. The assay included internal controls for quality assurance and laboratory staff was blinded to clinical findings. A positive result was defined as detection of at least one high-risk HPV type.

## Histopathology:

Women with abnormal cytology (≥ASC-US) or positive HPV results underwent colposcopy-guided biopsy. Tissue samples were processed and reviewed at BMC, Sagar by two pathologists blinded to cytology and HPV results. Lesions were graded as CIN1, CIN2, CIN3, or invasive carcinoma. CIN^2+^ was considered the study endpoint.

## Statistical analysis:

Data were analyzed using IBM SPSS version 25.0 (Chicago, USA). Sensitivity, specificity, positive predictive value (PPV), negative predictive value (NPV) and overall accuracy were calculated with 95% confidence intervals. Receiver operating characteristic (ROC) curves were plotted and the area under the curve (AUC) was calculated to assess diagnostic performance. The Chi-square test was used to compare proportions, with a p-value <0.05 considered statistically significant.

## Results:

A total of 400 women were included in the study. The average age was 42.5 years (±10.2). Most women were multiparous (65%) with a mean parity of 2.3. Rural residence was common, seen in 70% of participants. More than half (55%) had an early marriage before the age of 21. The most frequent symptom was abnormal vaginal discharge (60%), followed by post-coital bleeding (20%) and other complaints such as intermenstrual bleeding (20%) (Table 1). Out of 400 women, 250 underwent colposcopy-guided biopsy due to abnormal cytology or positive HPV results. On histopathology, 150 cases (37.5%) were reported as normal or inflammatory. Low-grade lesions (CIN1) were found in 200 women (50%). High-grade lesions (CIN2+) were detected in 50 cases (12.5%), which included 30 CIN2, 15 CIN3 and 5 invasive squamous cell carcinomas (Table 2). When compared with histopathology as the gold standard, HPV DNA testing showed the highest sensitivity (90%) for detecting CIN2+ lesions. Cytology had a lower sensitivity of 75%. Co-testing gave the highest sensitivity (95%), but this came with a much lower specificity (25%). Overall accuracy was better for HPV DNA testing (63.8%) than for cytology (48.8%) or co-testing (35%). The area under the ROC curve (AUC) was also highest for HPV DNA testing (0.75), suggesting superior diagnostic performance (Table 3). The ROC curve analysis (Figure 1 - see PDF) demonstrated superior diagnostic performance of HPV DNA testing compared to cytology and co-testing. Among women who tested positive for HPV, genotype 16 was the most common. It was found in nearly two-thirds of CIN2+ lesions. HPV18 was the next most frequent, followed by HPV52 and HPV58. Together, HPV16 and HPV18 accounted for about 70% of high-grade lesions, a pattern consistent with both global and Indian data. The contribution of other high-risk types such as HPV31, 33, 45 and 66 was smaller but still relevant. These findings show that most precancerous lesions in this population are linked to genotypes already targeted by current vaccines.

## Discussion:

This study showed that HPV DNA testing was more sensitive and accurate than cervical cytology for detecting CIN2+ lesions in women from Central India. Our results are consistent with large international studies that demonstrated HPV DNA testing to be more effective than cytology in identifying high-grade lesions [7]. The higher sensitivity of HPV DNA testing makes it a strong option for primary screening, especially in populations with a high burden of disease. Co-testing, which combines cytology and HPV testing, detected slightly more cases than HPV alone. However, its specificity was much lower. This means more women would be referred for colposcopy even when serious disease was not present. A recent network meta-analysis also found that co-testing is the most sensitive approach, but it sacrifices specificity, which is a concern in low-resource settings where unnecessary referrals increase costs and workload [8]. Histopathology confirmed the final diagnosis of CIN2+ lesions, reinforcing its role as the gold standard. In our study, only 12.5% of women had CIN2+ lesions, while most cases were either low-grade or normal. This finding reflects real-world screening programs, where the majority of screen-positive women do not have high-grade disease. Genotype analysis in our cohort revealed that HPV16 was the most common high-risk type detected, followed by HPV18 and a few other hrHPV types. These findings are in line with previous Indian studies, which showed HPV16 present in more than 80% of cervical cancer cases and HPV18 as the second most frequent type [9, 10]. A study from eastern India also reported similar results and estimated that the current 9-valent vaccine could cover over 90% of high-risk genotypes in India [11, 12]. Globally, HPV16 and 18 together are responsible for nearly 70-75% of cervical cancers [13]. This strengthens the argument for introducing and scaling up HPV vaccination programs in India. Our study has some limitations. First, it included only symptomatic women, which may overestimate the prevalence of abnormalities compared to general population screening. Second, we did not assess cost-effectiveness or acceptability of HPV testing, which are important for national program planning. Despite these limitations, the findings highlight that HPV DNA testing is a more reliable method for detecting cervical precancer than cytology. Future studies in India should look at larger community-based samples, evaluate the role of self-sampling for HPV testing and assess the long-term impact of integrating HPV-based screening into national programs.

## Conclusion:

hrHPV DNA testing is more sensitive and accurate than cytology for detecting CIN2+ lesions and is therefore suitable as a primary screening tool in resource-limited regions. Histopathology remains essential for definitive diagnosis, while HPV16/18 predominance underscores the importance of vaccination. Combining HPV testing with targeted cytology triage could strengthen cervical cancer prevention programs.

## Funding:

This research received no specific grant from any funding agency in the public, commercial, or not-for-profit sectors.

## Authors' contributions:

All authors read and approved the final manuscript.

## Atul Jain:

study conception, histopathology supervision and principal investigator.

## Mamta Meena:

data interpretation, manuscript preparation.

## Shiny Vincent:

laboratory support, HPV testing validation.

## Iqra Sheikh:

clinical pathology reporting, data preparation, preliminary analysis.

## Surabhi Sharma:

literature review, data compilation, drafting manuscript.

## Kuldeep Singh:

microbiological support, final editing.

## Rajdeep Paul:

project coordination, HPV methodology review, final editing.

## Figures and Tables

**Table 1:** Demographic and clinical features of participants (n = 400)

**Variable**	**Value**
Mean age (years)	42.5 ±10.2
Multiparous	260 (65%)
Mean parity	2.3
Rural residence	280 (70%)
Early marriage (<21 years)	220 (55%)
Abnormal discharge	240 (60%)
Post-coital bleeding	80 (20%)
Other symptoms	80 (20%)

**Table 2:** Histopathology results (n = 400)

**Diagnosis**	**Number (%)**
Normal / Inflammation	150 (37.5%)
CIN1 (LSIL)	200 (50.0%)
CIN2	30 (7.5%)
CIN3	15 (3.8%)
Invasive carcinoma (SCC)	5 (1.2%)
Total CIN2+	50 (12.5%)

**Table 3:** Diagnostic performance of screening tests for CIN2+

**Test Method**	**Sensitivity (95% CI)**	**Specificity (95% CI)**	**PPV (95% CI)**	**NPV (95% CI)**	**Accuracy (%)**	**AUC (95% CI)**
Cytology (LBC)	75.0 (60.4-86.4)	45.0 (39.2-50.9)	17.5 (12.5-23.5)	91.5 (85.8-95.4)	48.8	0.60 (0.52-0.68)
HPV DNA	90.0 (78.2-96.7)	60.0 (54.0-65.8)	24.3 (18.2-31.3)	97.5 (94.1-99.2)	63.8	0.75 (0.68-0.82)
Co-testing	95.0 (83.1-99.4)	25.0 (20.0-30.5)	15.8 (11.7-20.7)	97.2 (90.3-99.7)	35	0.60 (0.53-0.67)
